# Alzheimer's Dementia due to Suspected CTE from Subconcussive Head Impact

**DOI:** 10.1155/2018/7890269

**Published:** 2018-07-31

**Authors:** Shauna H. Yuan, Sonya G. Wang

**Affiliations:** ^1^Department of Neurosciences, University of California, San Diego, La Jolla, CA 92093, USA; ^2^Department of Neurology, University of Minnesota, Minneapolis, MN 55455, USA

## Abstract

Chronic traumatic encephalopathy (CTE) has been receiving increasing attention due to press coverage of professional football players. The devastating sequelae of CTE compel us to aim for early diagnosis and treatment. However, by current standards, CTE is challenging to diagnose. Clear clinical diagnostic criteria for CTE have not been established. Only recently, pathological diagnostic criteria have been recognized, but postmortem diagnosis is too late. Reliable biomarkers are not available. By imaging criteria, cavum septum pellucidum has been the only consistent identifiable MRI finding. Because of the imprecise nature of diagnosis based on clinical suspicion, physicians must become cognizant of the broad spectrum of presentations of CTE. With this awareness, appropriate workup can be initiated. CTE can present with early symptoms of emotional changes or late symptoms with memory decline and dementia. Here we present an unusual case of a patient with Alzheimer's disease secondary to suspected CTE that stems from subconcussive head impacts presenting with severe memory and MRI changes. Clinicians should be aware of this presentation and consider CTE in their differential diagnoses while undergoing workup of memory disorders.

## 1. Introduction

Chronic traumatic encephalopathy (CTE) is the long-term consequence of brain injury, due to repetitive brain trauma. It is associated with collision sport athletes, military personnel, domestic violence, and head banging behavior. The incidence for concussion is high, affecting between 1.6 and 3.8 million people per year [1, 2]. The prevalence for CTE is less clear; however, a recent study suggests that up to 87% of the brains from former football players displayed CTE pathology [3].

CTE was first described as “punch drunk” syndrome in retired boxers [4]. Later it was clinically labelled “dementia pugilistica” or “boxer's dementia” [1, 5]. Patients can present with behavioral, motor, and cognitive symptoms. Behavioral manifestations are frequently seen earlier in the disease, including symptoms of depression, emotional lability, apathy, aggression, suicidality, and apathy. Spasticity, tremor, ataxia, dysarthria, and incoordination are associated motor symptoms. Cognitively, patients develop impaired attention and concentration, memory deficits, and dementia. Clinical diagnostic criteria based on the numerous manifestations aforementioned have been proposed for research purposes only [6–8] and overall are extremely vague. Definitive diagnosis of CTE is by examination of the postmortem tissue. Diagnostic criteria on tissue pathology include a dot-like distribution of phosphorylated tau aggregated in neurons, astrocytes, and cell processes in perivascular spaces within the depth of the cortical sulci [9]. Phosphorylated tau aggregates in cortical layers II and III and hippocampal CA2 and CA4 regions are supportive of diagnosis of CTE. TDP43 and amyloid plaques can be associated with CTE.

There is no reliable biomarker for CTE. Imaging of CTE shows numerous varied findings, with only cavum septum pellucidum being a consistent finding. In a study of boxers, brain MRI showed hippocampal atrophy and cavum septum pellucidum, periventricular white matter changes, and cortical atrophy [10, 11]. Studies involving Diffusion Tensor Imaging (DTI) have shown abnormalities in the deep white matter and cortical gray matter in boxers [12, 13]. Cerebral perfusion studies in boxers have shown increased abnormalities in the boxer group in multiple regions scattered throughout the cortex compared to control [14]. FDG-PET imaging shows decreased uptake bilaterally in the posterior cingulate cortex, parietooccipital and frontal lobes, and cerebellum in boxers [6]. PET tracers such as THK5317, THK5351, AV1451, PBB3, and [F-18] FDDNP can be utilized to trace tau deposition; however the usage is still experimental and the efficacy of using these to diagnose CTE still needs to be evaluated [7, 8, 15, 16].

Emerging evidence shows that repetitive subconcussive head impacts correlate best with CTE [17–19], suggesting that subconcussive head injuries are harmful. Subconcussion is defined by impact to the head, which causes brain changes similar to concussion, without the acute clinical symptoms [20]. Thus, CTE can be missed as a diagnosis since patients and families do not typically recall these subtler events without loss of consciousness. Studies have shown that subconcussive impact leads to brain changes [21]. Cognitive, functional, and biochemical changes occur in the brains of contact sport players, without a clearly diagnosed concussion [22–24]. Neuropathologically confirmed CTE cases all had previous history of repetitive brain trauma [25]. Repetitive brain trauma leads to neurodegenerative diseases, such as Parkinson's disease, frontotemporal lobar degeneration (FTLD), and Alzheimer's dementia [26, 27].

Currently management of CTE is symptomatic treatment of the neuropsychiatric or cognitive symptoms. Because many of the patients present at a younger age, while they are still working, diagnosis of CTE is critical for treatment, counseling, and prognosticating. Unless physicians consider CTE in their differential diagnosis, these younger patients cannot obtain their necessary early treatment interventions. In addition, the proper diagnosis is particularly important for caregivers as they will require guidance in terms of planning and coping with this devastating disease.

Increasing the awareness of these clinical presentations for the clinicians is key to facilitating the appropriate workup and management. Here we present a case of presumed CTE with a former football player, who suffered from repetitive subconcussive brain impact. He presented with severe memory, cognitive deficits, and significant MRI changes and thus was diagnosed with Alzheimer's disease secondary to suspected CTE.

## 2. Case Presentation

Patient was a 54-year-old right-handed male, former professional football player. He first developed memory problems at the age of 46. Initially, he seemed more forgetful. The onset and the progression of the short-term memory problem were gradual over about eight years. He always did his own finances in the past. However at the age of 46, he started spending money more irrationally and was not paying the bills on-time. He repeated questions, sometimes even just a few minutes later. He had trouble learning new information. He could not manage his own calendar. He has become dependent on the GPS to get around. Patient had become less social. He did not have depressed mood; however, he had become more irritable and more easily angered. He had no behavioral issues. His activities of daily living (ADLs) were intact.

Patient started playing football when he was age 7 or 8. He played football in high school and college and then professionally. He played football for total of 23 years. Although he never lost consciousness, he experienced brief moment of flashes. This type of head injury averaged 3-4 times per game. There is no family history of dementia.

**Figure 1:**
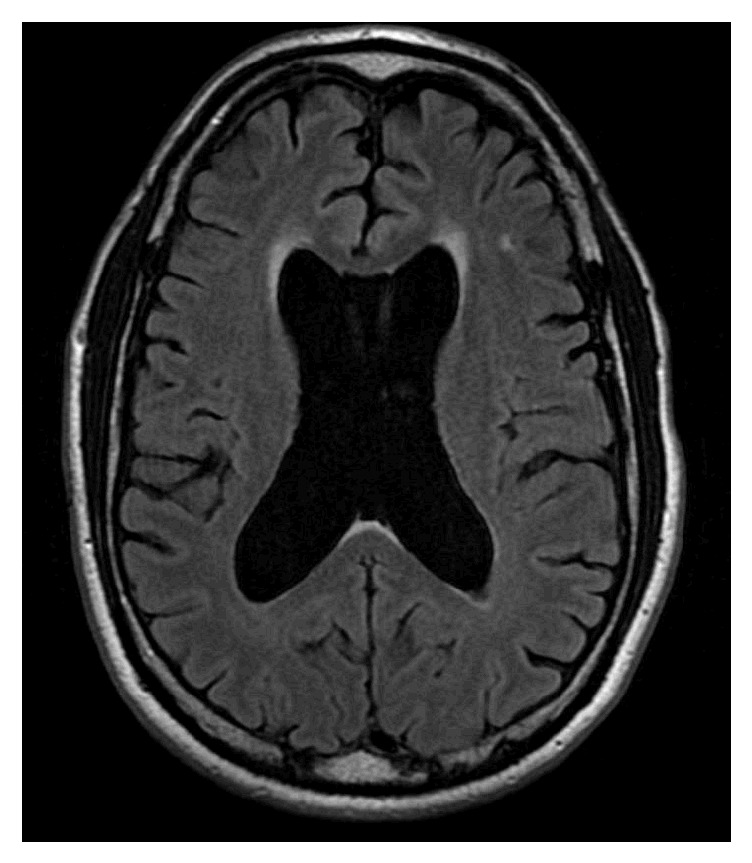
**MRI reveals changes related to possible CTE**. Axial image reveals both cortical and subcortical atrophy, enlarged lateral and third ventricle, and cavum septum pellucidum.

His mini-mental status exam (MMSE) was 24/30, and the clinical dementia rating (CDR) was 1. On neuropsychological testing, he had significant impaired verbal and nonverbal learning, recall, and recognition with rapid forgetting (more than two standard deviations). Patient's MRI showed cortical and subcortical atrophy, enlarged ventricles, and cavum septum pellucidum (Figure 1). The hippocampal volume was below 5 percentile and the inferior lateral ventricle volume was greater than 95 percentile. His diagnosis was major neurocognitive disorder, likely Alzheimer's disease due to CTE.

## 3. Discussion

This case was diagnosed as early onset Alzheimer's dementia with presumed CTE, for insidious gradual onset of memory impairment. Findings in the neuropsychological testing are consistent with AD. However, history of playing professional football and no other risk factors for AD suggest that CTE is the underlying cause of his memory impairment and MRI findings. Because there are no established clinical diagnostic guidelines for CTE, the diagnosis for CTE can only be proposed. Patient had low hippocampal volume, which fits the profile for AD [28]. The history of subconcussive head impact and the presence of cavum septum pellucidum are supportive of CTE. AD pathology may coexist with CTE; therefore, it is important for clinicians to recognize that these two conditions can overlap. In neuropathological study, amyloid plaque coexists in 50% of CTE pathology [29]; therefore, beta-amyloid deposition is possibly induced by CTE pathology.

In contrast to early presentation of neuropsychological symptoms [30], this patient presented with memory changes and cognitive impairment later in life that developed into dementia. This presentation is consistent with the current understanding that neuropsychological symptoms of early and cognitive impairment/dementia occur late in the course of CTE [31]. The degree of ventricular enlargement was significantly large for his age, consistent with advanced stage of CTE [32].

The anatomical distribution of hyperphosphorylated tau deposition suggests possible breakdown of blood brain barrier and inflammatory response during traumatic brain injury. Phosphorylated tau appearance later in disease in area other than the perisulcal space suggests that possible tau propagation. Mechanism for CTE has been proposed to involve three stages of pathological changes, including an acute/subacute state, followed by chronic/static state and then neurodegeneration [33].

In conclusion, we suggest that the clinicians should be aware of subconcussion contributing to CTE. They should include CTE in their differential diagnosis when evaluating patients with memory impairment and dementia and recognize that Alzheimer's and CTE can coexist.
